# Unilateral tactile agnosia as an onset symptom of corticobasal syndrome

**DOI:** 10.3389/fnhum.2024.1401578

**Published:** 2024-07-25

**Authors:** Laura Facci, Stefania Basilico, Manuela Sellitto, Giorgio Gelosa, Martina Gandola, Gabriella Bottini

**Affiliations:** ^1^Department of Brain and Behavioral Sciences, University of Pavia, Pavia, Italy; ^2^Cognitive Neuropsychology Centre, ASST Grande Ospedale Metropolitano Niguarda, Milan, Italy; ^3^NeuroMI, Milan Center for Neuroscience, Milan, Italy

**Keywords:** tactile agnosia, corticobasal syndrome, hyloagnosia, morphoagnosia, tactile object recognition

## Abstract

Tactile agnosia is the inability to recognize objects via haptic exploration, in the absence of an elementary sensory deficit. Traditionally, it has been described as a disturbance in extracting information about the physical properties of objects (“apperceptive agnosia”) or in associating object representation with its semantic meaning (“associative agnosia”). However, tactile agnosia is a rare and difficult-to-diagnose condition, due to the frequent co-occurrence of sensorimotor symptoms and the lack of consensus on the terminology and assessment methods. Among tactile agnosia classifications, hyloagnosia (i.e., difficulty in quality discrimination of objects) and morphoagnosia (i.e., difficulty in shape and size recognition) have been proposed to account for the apperceptive level. However, a dissociation between the two has been reported in two cases only. Indeed, very few cases of pure tactile agnosia have been described, mostly associated with vascular damages in somatosensory areas, in pre- and postcentral gyrus, intraparietal sulcus, supramarginal gyrus, and insular cortex. An open question is whether degenerative conditions affecting the same areas could lead to similar impairments. Here, we present a single case of unilateral right-hand tactile agnosia, in the context of corticobasal syndrome (CBS), a rare neurodegenerative disease. The patient, a 55-year-old woman, initially presented with difficulties in tactile object recognition, apraxia for the right hand, and an otherwise intact cognitive profile. At the neuroimaging level, she showed a lesion outcome of a right parietal oligodendroglioma removal and a left frontoparietal atrophy. We performed an experimental evaluation of tactile agnosia, targeting every level of tactile processing, from elementary to higher order tactile recognition processes. We also tested 18 healthy participants as a matched control sample. The patient showed intact tactile sensitivity and mostly intact hylognosis functions. Conversely, she was impaired with the right hand in exploring geometrical and meaningless shapes. The patient’s clinical evolution in the following 3 years became consistent with the diagnosis of CBS and unilateral tactile apperceptive agnosia as the primary symptom onset in the absence of a cognitive decline. This is the third case described in the literature manifesting morphoagnosia with almost completely preserved hylognosis abilities and the first description of such dissociation in a case with CBS.

## 1 Introduction

Tactile object recognition (TOR) is a complex ability that requires the detection of elementary sensory information (e.g., touch, pain, temperature, vibration, and proprioception), the processing of characteristics of objects, such as weight, texture, size, and shape, and the integration of this information into a coherent representation, with a subsequent association of this sensory representation with semantic knowledge (Veronelli et al., 2014; Reed and Ziat, 2018).

Historically, the process of object recognition has been divided into two stages: (1) the perception of physical features of objects, or “apperceptive”, and (2) the association of the perceptive representation with semantic memory, i.e., “associative level” (Lissauer, 1890). Although this distinction was primarily proposed for the visual modality (Lissauer, 1890), some authors argued that it could also be applied to the tactile modality, albeit with an additional distinction at the perceptual level (Bottini et al., 1991; Reed et al., 1996). Particularly, Delay (1935) postulated a distinction between *hylognosis*, i.e., the ability to discriminate the qualities of objects such as weight, texture, and thermal properties, and *morphognosis*, i.e., the ability to discriminate the shape and size of objects.

Existing cognitive models of TOR are mainly derived from the clinical observation of patients with difficulties in recognizing objects via haptic exploration (Caselli, 1991; Endo et al., 1992). This impairment is called tactile agnosia, a modality-specific deficit characterized by the inability to recognize objects by touch in the absence of primary sensory impairment (Claparède, 1899; Endo et al., 1992). However, although agnosia has been extensively investigated for the visual modality and, to some extent, for the acoustic one (Vignolo et al., 1997; Simons and Lambon Ralph, 1999; Riddoch and Humphreys, 2003; Burns, 2004; Garrido et al., 2018; Miceli and Caccia, 2023), haptic recognition mechanisms are still poorly understood (Bottini et al., 1995; Bohlhalter et al., 2002). The difficulty in investigating tactile agnosia derives from the rarity of this disorder and the lack of consensus on both the semantic labelling, as different terms have been proposed to define the same impairment, and the adopted methods of investigation (Endo et al., 1992; Reed et al., 1996; Saetti et al., 1999; Kubota et al., 2017). For example, in the literature, the term “morphoagnosia” has been used to indicate a difficulty in shape and size discrimination, which does not necessarily preclude real object identification (Endo et al., 1992; Reed et al., 1996; Saetti et al., 1999; Kubota et al., 2017), distinct from “hyloagnosia,” i.e., the inability to discriminate object’s qualities. Morphoagnosia together with hyloagnosia accounts for Lissauer’s apperceptive level. However, a double dissociation between hyloagnosia and morphoagnosia has never been reported, and the presence of morphoagnosia without hyloagnosia has been described only in two cases (Saetti et al., 1999; Kubota et al., 2017). The impairment in shape recognition has also been referred to as “astereognosis,” when the difficulty is secondary to an impairment in spatial discrimination (Carlson and Brooks, 2009). On the other hand, an impairment in real object recognition in the absence of hyloagnosia and morphoagnosia has been referred to as “tactile asymbolia” (Caselli, 1991), accounting for Lissauer’s associative level, and to be distinguished from tactile aphasia (i.e., the inability to name tactually identified objects; Geschwind, 1965; Endo et al., 1992). Considering the different clinical manifestations of TOR impairments, some authors have even questioned the existence of “pure” tactile agnosia, claiming that difficulties in haptic perception appeared only secondary to elementary, sensorimotor, or cognitive disorders (Bay, 1944; Semmes, 1965). Nevertheless, despite the difficulty in disentangling the elementary somesthetic deficits from the more cognitive processes of tactile recognition, the few cases that systematically explored the impairments seem to support the existence of tactile agnosia *per se*, disentangling agnosia from tactile aphasia (Endo et al., 1992) and from general spatial impairments (Reed et al., 1996).

Although tactile agnosia usually manifests on one hand (Platz, 1996; Reed et al., 1996; Valenza et al., 2001; Estañol et al., 2008; Hömke et al., 2009; Veronelli et al., 2014; Kubota et al., 2017), the brain damage may be also bilateral (Endo et al., 1992; Saetti et al., 1999).

Depending on the clinical features of tactile agnosia, the neural substrate involves several cortical regions: in particular, the secondary somatosensory area (SII), the inferior parietal cortex, the insula (Reed et al., 1996; Bohlhalter et al., 2002), the pre- and postcentral gyri (Crutch et al., 2005; Hömke et al., 2009; Kubota et al., 2017), the supramarginal gyrus, the intraparietal sulcus, and the superior parietal lobule (Endo et al., 1992; Platz, 1996; Stoeckel et al., 2003; Hömke et al., 2009; Veronelli et al., 2014). Tactile agnosia is usually due to brain events of vascular origin (Caselli, 1991; Hömke et al., 2009) and in one reported case due to a meningioma (Platz, 1996).

However, an open question is whether other types of brain damage, including degenerative conditions, can lead to similar TOR impairments. In particular, corticobasal syndrome (CBS), a degenerative disease that clinically presents heterogeneous cognitive and/or motor disturbances (Armstrong et al., 2013), is characterized by asymmetric cerebral atrophy, affecting different brain areas depending on the underlying pathology that causes the syndrome, such as Alzheimer’s disease (AD), progressive supranuclear palsy (PSP), or corticobasal degeneration (CBD) (Boeve et al., 1999; Whitwell et al., 2010). All these pathological classifications share a pattern of gray matter loss in the supplementary motor area (SMA), insula, premotor cortex, and parietal lobe (Whitwell et al., 2010), areas that are also involved in TOR.

Interestingly enough, TOR impairments usually are not formally and extensively assessed in CBS, although the presence of cortical sensory loss is one of the main clinical features associated with this pathological condition (Bassetti et al., 1993; Mathew et al., 2012; Matsuda et al., 2020). The term “cortical sensory loss” falls within the realm of somatosensory disorders, but its definition is vague. Some authors describe it as the co-occurrence of astereognosis, agraphesthesia, and loss of position sense (Bassetti et al., 1993), whereas others include the extinction of double tactile stimuli (Belfor et al., 2006). Additionally, some authors define it as “tactile inattention” and an impairment in the two-point discrimination task (Misra et al., 1997). Others incorporate, in the assessment of the cortical sensory loss, also the evaluation of pain and temperature discrimination (Kim, 2007; Armstrong et al., 2013; Chahine et al., 2014).

Actually, to the best of our knowledge, only one study tried to provide a more precise characterization of these deficits (Matsuda et al., 2020), interpreting cortical sensory loss as a “somatosensory dysfunction” and investigating tactile localization, weight and texture perception, letter, and object recognition. In the study by Matsuda et al., the authors found difficulties in patients with CBS in the two-point discrimination task and object naming. However, their study did not properly explore the presence of morphoagnosia through the test of recognition of geometrical and meaningless shapes.

Here, we systematically investigated the tactile object recognition competence of a patient who came to our attention for a subjective progressive worsening in her right-hand sensitivity associated with difficulty in haptically recognizing objects with the right hand. She was administered an extensive experimental testing to assess the three levels of tactile processing as proposed by Caselli (1991), i.e., basic somatosensory functions, intermediate level (hylognosis and morphognosis), and real object recognition.

## 2 Materials and methods

### 2.1 Participants

#### 2.1.1 Patient CP

CP is a right-handed 55-year-old woman, still an active lawyer. In July 2020, she was admitted to the Department of Neurology at Niguarda Hospital for subjective right-hand and arm dysesthesia. The patient underwent a CT scan that showed an oligodendroglioma in the right posterior parietal parasagittal area, surgically removed in the same year. CP came to our attention at the Cognitive Neuropsychology Centre of the same hospital in 2021, complaining of being unable to find objects inside her bag when searching with her right hand. At the neurological examination, she was cooperative and well-oriented to person, place, and time. She was hypomimic with blinking reduction. No language impairment was detected. The examination also highlighted right-hand tactile hypoesthesia with impairment in fine movements, ideomotor apraxia to the right hand with fine-amplitude action tremor, and plastic rigidity of upper limbs more evident on the right side with trochlea at the wrists; no myoclonus nor dystonia was observed. Walking was preserved with arm swing reduced on the left and absent on the right; the pull test was negative. An MRI examination documented a postsurgical liquor-filled cavity in the posterior parasagittal right parietal region, along with a diffuse hyperintense signal in the left frontoparietal cortical–subcortical area, associated with left focal atrophy of the convolutions, particularly in the precentral and postcentral gyri, as well as a marked enlargement of the corresponding cortical sulci. A DaTSCAN and an 18-FDG SPECT were both negative.

At the first neuropsychological visit in 2021, at the 6-month postsurgical follow-up, we administered an extensive neuropsychological assessment (see Table 1) and found right-hand ideomotor apraxia (Tessari et al., 2013) and difficulties in right-hand haptic object recognition measured through the stereognosis subtest of the Nottingham Sensory Assessment (Lincoln et al., 1998). As the standardization of this subtest for the Italian population is missing, we referred to Spanish normative data (Zamarro-Rodríguez et al., 2021), for which only the right-hand score was deficient. It is important to point out that the clinical evaluation showed a slowdown and clumsiness of the movements in the right hand. Left-hand object recognition, visual object recognition, and other cognitive functions were preserved. Therefore, the haptic abilities were investigated in more detail.

**Table 1:** CP’s score at the neuropsychological assessment.

Test	CP’s adjusted score
**General cognitive status**	
MMSE (Measso et al., 1993)	30 (30)
Activities of daily living (Katz, 1983)	6 (6)
Instrumental activities of daily living (Katz, 1983)	8 (8)
**Language**	
Verbal fluency (Novelli et al., 1986)	42.54
Semantic fluency (Novelli et al., 1986)	43.71
Picture-naming test (Catricalà et al., 2013)	48 (48)
**Verbal memory**	
Short story recall (Novelli et al., 1986)	13.5 (28)
Immediate recall	10 (28)
Delay recall	17 (28)
Rey AVLT (Carlesimo et al., 2014)	
Immediate recall	37.2 (60)
Delay recall	6 (15)
Digit span (Monaco et al., 2013)	6.66 (9)
Digit span backward (Monaco et al., 2013)	5.65 (8)
**Visuospatial memory**	
Rey–Osterrieth complex figure (Carlesimo et al., 2002)	
Immediate recollection	36 (36)
Delayed recollection	14.5 (36)
Corsi span (Monaco et al., 2013)	6.62 (9)
Corsi span backward (Monaco et al., 2013)	3.82 (8)
**Attention and executive functions**	
**Trail making test** (Giovagnoli et al., 1996)	
A	45.90″
B	88.25″
Stroop test (Caffarra et al., 2002)	
Time	15.84″
Errors	0
Symbol digit modality test (Nocentini et al., 2006)	66 (110)
Cancellation test (Uttl and Pilkenton-Taylor, 2001)	53 (53)
**Ideomotor apraxia**	
Right arm apraxia (Tessari et al., 2013)	55^*^ (72)
Left arm apraxia (Tessari et al., 2013)	65 (72)
**Object recognition**	
Stereognosis subtest of NSA (right hand)	13^*^ (20)
Stereognosis subtest of NSA (left hand)	19 (20)

Scores are adjusted based on Italian normative data. MMSE, Mini-Mental State Examination; Rey AVLT, Rey Auditory Verbal Learning Test; NSA, Nottingham Sensory Assessment. ^*^Impaired. () maximum score for the test when available.

CP underwent clinical, neuropsychological, and neuroimaging (MRI) follow-up every year (2021–2023). In 2023, she also repeated both the experimental evaluation for tactile object recognition and the DaTSCAN.

This study was carried out in accordance with the recommendations of the Ethical Committee Milano Area 3, and the patient signed the informed consent.

#### 2.1.2 Control participants

We recruited a matched control sample of 18 healthy female participants (mean age = 54.5 ± 5.33; mean years of education = 16.22 ± 3.34). They were all right-handed with normal tactile capabilities and no self-reported neurological or psychiatric pathologies. They all gave written informed consent according to procedures approved by the Ethical Committee of the University of Pavia.

### 2.2 Experimental and clinical behavioral examination

The clinical and experimental assessment was inspired by the classification proposed by Caselli (1991), which distinguishes between basic somatosensory functions, intermediate somatosensory functions, and real object recognition. We devised *ad hoc* tests to investigate all these levels. All tests were administered while the patient and control participants were blindfolded.

#### 2.2.1 Basic somatosensory functions

##### 2.2.1.1 Tactile detection

Single light touches on the dorsal surface were administered on the patient’s left or right hand. The patient was asked to tell in which hand she perceived the stimulus, while keeping her eyes closed. In total, 15 stimuli were randomly delivered to the left and the right hand and mixed with 10 catch trials. In a second task, the single tactile stimuli were mixed with 15 double simultaneous touches delivered on both hands to assess the presence of tactile extinction.

##### 2.2.1.2 Two-point discrimination

We administered the two-point discrimination task (Weber, 1835; Louis et al., 1984) to assess the ability to perceive two points simultaneously delivered to the skin as separated (i.e., a measure of tactile acuity). The two tips of a digital caliper (*Metrica 10745*) were applied on the index finger and the palm of the hand while the patient was asked to judge whether she felt one or two points. Distance between the two points started from 25 mm and decreased by 5 mm from 25 to 15 mm, and then by 1 mm from 14 to a minimum of 1 mm. A standardized correction scale is provided for this task, which was not administered to the control sample (Shimokata and Kuzuya, 1995).

##### 2.2.1.3 Semmes–Weinstein test

The sensory evaluation of the tactile detection threshold was performed on both hands with the four Semmes–Weinstein monofilaments (2.83 mm, 3.61 mm, 4.31 mm, and 6.45 mm) (*Touch Test^®^ Sensory Evaluators Hand Kit, North Coast Medical, Inc.*); two touches over every finger (upper fingertip and lower finger) and one touch on the palm were administered asking the patient whether she could feel the stimulation. A standardized correction scale is provided also for this test (Bell-Krotoski et al., 1995).

##### 2.2.1.4 Finger dexterity (finger tapping movement)

The patient was asked to rapidly move the fingers of the hands in progression, tapping on the table, first from thumb to little finger and then in the opposite direction, twice. The sequence of finger movements was shown in advance by the experimenter, who qualitatively assigned 0 points if the patient was not able to imitate the movement, 1 point if the movement was slow, and 2 points if it was correctly executed.

##### 2.2.1.5 Perception of pain

The patient was stimulated on the back of the hand with a non-harmful painful stimulus (toothpick) or a neutral stimulus (cotton pad) for approximately 1 s, and she had to indicate whether the perceived stimulus was painful or neutral; 10 trials for each hand were administered.

##### 2.2.1.6 Temperature discrimination

A test tube containing warm (approximately 40°C) or cold (approximately 10°C) water was placed on the back of the hand for 2 s, 10 trials for each hand. The patient had to indicate whether the perceived stimulus was warm or cold.

##### 2.2.1.7 Perception of vibration

A neurological tuning fork was placed on the back of the patient’s hand by asking her to state whether she felt or not the vibration; 10 trials (five vibration and five no-vibration) were administered.

##### 2.2.1.8 Proprioception

The examiner moved the middle finger of the patient’s hand up or down in relation to the other fingers, and the subject was asked to report, while keeping the eyes closed, whether the finger was moved up or down; 10 trials for each hand were administered.

#### 2.2.2 Intermediate somatosensory functions: hylognosis and morphognosis

##### 2.2.2.1 Weight discrimination

The patient was asked to compare four plastic balls with different weights with a target ball: two balls were heavier, and two were lighter than the target. A ball was placed on the patient’s hand immediately after the target. The task was repeated twice. The target was re-presented before every comparison to prevent working memory overload.

##### 2.2.2.2 Texture discrimination

The patient was asked to compare four grades of sandpaper with a target sandpaper; two smoother and two rougher pieces of sandpaper than the target were presented twice, placing them one at a time on the same hand immediately after the target.

##### 2.2.2.3 Naming of materials

The patient was presented with 10 squared pieces made of different materials: plastic, metal, wood, glass, foam rubber, paper, rubber, cotton, polystyrene, and fabric. She was asked to explore the material as long as she wanted and to name it. In the controls sample, denomination of materials was made with one hand only, to prevent any learning effect. Half of the control sample did the task with the left hand and the other half with the right hand.

##### 2.2.2.4 Size discrimination

The patient was asked to compare four polyester balls with the same weight and texture but different in size, with a target ball; two smaller and two bigger balls than the target were presented twice, placing them one at a time on the same hand, immediately after the target.

##### 2.2.2.5 Two-dimensional figures

In total, eight wooden-made geometrical shapes (rectangle, square, pentagon, circle, cross, star, triangle, and rhombus) were placed on the plasticine allowing to hold them still, with only the edges emerging by a few millimeters to favor two-dimension exploration. The patient was presented with eight pairs of these shapes (four identical and four different pairs) and asked to discriminate whether the second stimulus in the pair was the same or different from the first one.

##### 2.2.2.6 Geometrical shapes

The same eight geometrical figures of the previous task were given to the patient, without plasticine, asking her to explore them in three dimensions with her whole hand, for eight pairs of same/different comparisons.

##### 2.2.2.7 Meaningless shapes

**Figure 1:**
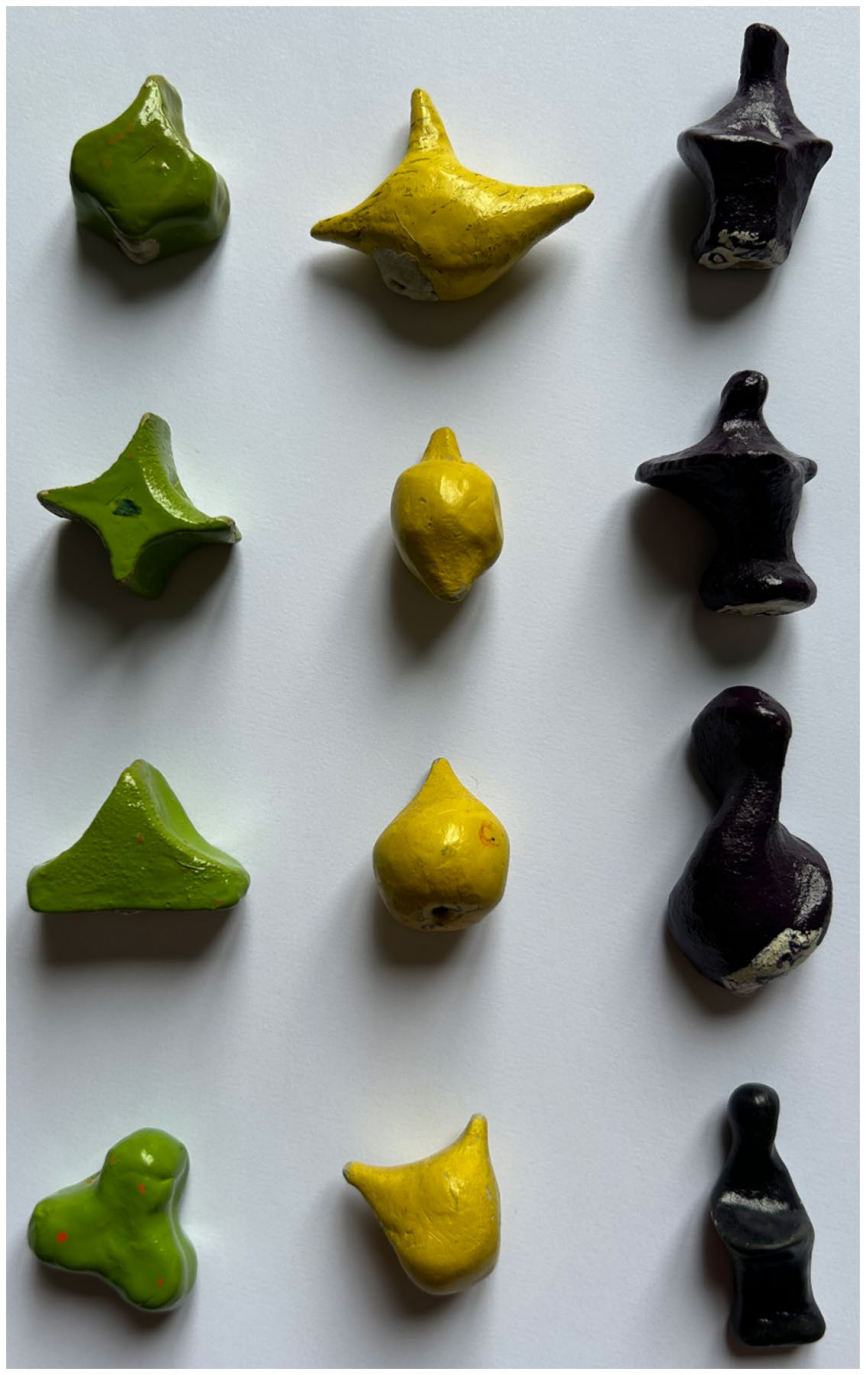
Meaningless shapes used in the test for morphoagnosia (Bottini et al., 1991).

We used a selection of the meaningless shapes used by Bottini et al. (1991). The patient was asked to compare 12 couples of the same or different shapes. The shapes were identical in size (5 cm × 3 cm), texture, and weight (20 g) (Figure 1). The first stimulus was placed in the patient’s hand, as a reference target, and she was allowed to manipulate it as long as she wanted. Then, the reference target was removed, and, immediately after, the same identical object or a different one was placed in her hand, asking whether it was the same or different; three meaningless shapes were presented as reference targets, four times each one. As in the previous tasks, the target was re-presented before every comparison stimulus to prevent working memory overload.

#### 2.2.3 Real object recognition

##### 2.2.3.1 Nottingham sensory assessment—stereognosis subtest

This test involves the use of 10 real objects: a 2€ coin, a 50-cent coin, a pen, a pencil, a comb, a sponge, a scissor, a flannel, a cup, and a glass. The object was placed in the patient’s hand (max. 15 s), and she was asked to name or describe it; 2 points were given if the object was correctly named; 1 point if the patient was unable to identify it but still managed to describe some features; 0 point if the patient failed the identification. This test was administered only to the patient during the neuropsychological assessment and not to the control group.

##### 2.2.3.2 Naming of objects

The patient was asked to manipulate 12 real objects (lighter, brush, screwdriver, comb, fork, thimble, clothespin, teaspoon, sharpener, watch, and key) and to name them. After the tactile exploration with the hands, we asked the patient to open her eyes and name the objects. This test was not administered to control participants.

##### 2.2.3.3 Drawing an object after haptic exploration

To qualitatively investigate the patient’s mental representation of an object explored by touch, we asked the patient to manipulate a funnel with her eyes closed and to draw it before with the right hand and then with the left hand (Valenza et al., 2001). Then, the patient was asked to name the touched object. This test was not performed on control participants.

##### 2.2.3.4 Meaningful objects haptic recognition test

To further explore the right-hand impairment in tactile object recognition, we performed a meaningful object haptic recognition test, devising a comparison task that does not require naming the objects. The test was performed only with the right hand as the real object recognition with the left hand was intact (see Results section 3.3).

We used the same real objects as Bottini et al. (1991, 1995), with a modified task version. The patient was asked to compare 64 pairs of natural objects with the right hand and indicate whether the two touched objects were the same or different. The examiner explained to the patient that she would be presented with pairs of commonly used objects in which the second stimulus would have been: (i) an object with the same name, function, and shape as the first stimulus (*identity*: the same object was touched twice); (ii) an object with the same name and function as the first stimulus but with a different shape (*same category*: e.g., two kinds of comb); (iii) an object semantically related to the first stimulus (*semantic relation*: e.g., a pencil as first stimulus and an eraser as second one); and (iv) a different object not related in name, function, or shape to the first stimulus (*no relation*). In the first two conditions, when manipulating two identical objects (identity) or two objects with the same name and function but different shapes (same category), the patient had to answer “same”; in the latter two cases, when manipulating two semantic-related objects or non-related objects, she had to answer “different.” We presented 16 pairs of objects for each of the 4 categories (i) identity, (ii) same category, (iii) semantic relation, and (iv) no relation. For each trial, we registered exploration time and accuracy for the first and the second objects.

### 2.3 Procedure

The first experimental assessment was carried out in 2021 and repeated in 2023. Results of the first (2021) and the second (2023) experimental assessments were compared to the same group of control participants, who made the procedure once.

During the experimental assessment, CP’s right hand was always tested before the left hand, in order to prevent a learning effect. All tests were administered while the patient was blindfolded, and both hands were tested for each task, apart from the 64 comparisons of real objects, tested only for the right hand. The evaluation started with the assessment of basic somatosensory functions (see section 2.2.1), then of hylognosis and morphognosis (section 2.2.2), and ultimately of real object recognition (section 2.2.3). Because of the fatigability of the patient, we split the assessment into 3 sessions of 1.5 h each.

Control participants underwent the identical procedure as the patient, while blindfolded. They were submitted to the primary and intermediate tasks, including the meaningless shapes task, with both hands. Denomination of materials and comparison of real objects were performed with one hand only; half of the sample did the task with the right hand, and half with the left hand. The experimental session lasted approximately 2 h.

### 2.4 Statistical analysis

Patient’s performance was compared with that of the control sample using the modified Crawford–Howell *t*-test (Crawford and Garthwaite, 2002) implemented in the software program Singlims_ES (vers. 2010, available online: https://homepages.abdn.ac.uk/j.crawford/pages/dept/SingleCaseMethodsComputerPrograms.HTM).

The patient’s tactile detection, discrimination of pain, temperature, vibration, and proprioception, and comparison of weight, texture, size, geometrical shapes, meaningless shapes, and real objects were compared with those of the full 18-participant sample. On the other hand, the naming of materials after exploration with the left hand was compared with the results of the nine participants who did the task with the left hand, while the results of the right hand with the other half of the sample who did the task with the right hand.

Concerning the meaningful object haptic recognition test, we preprocessed exploration time within each participant by excluding data points over and below 2.5 standard deviations from each participant’s mean. This preprocessing was repeated for the first and the second stimulus, separately. We repeated this procedure for each participant and the patient before going to the second-level analysis (i.e., the patient–controls comparison).

## 3 Results

### 3.1 Processing of elementary sensory data

The patient’s response to the *Two-point discrimination* fell within the normal range [based on Tzourio-Mazoyer et al. (2002)] for both the index finger (two-point threshold distance: right finger = 1 mm; left finger = 4 mm) and the palm (two-point threshold distance: right palm = 13 mm; left palm = 9 mm). It is worth noting that the discrimination ability with the right palm (13 mm) was lower than with the left palm (9 mm), although remaining within the normal range.

CP also correctly completed the *Semmes–Weinstein* task: the fingertips sensibility fell within the normal range (2.83 mm perceived) for the upper left fingers and diminished for light touch in the left lower fingers (3.61 mm perceived); a diminished light touch sensibility was also found in the right hand for both upper and lower fingers. Normal sensibility emerged for right and left palms (2.83 mm perceived). Those results could be considered within the normal range based on the thresholds proposed by Bell-Krotoski et al. (1995). During the assessment of diadochokinesis, CP was able to complete the movements with both hands, and from a qualitative point of view, the movement of the right-hand fingers was clumsy as compared to the left-hand fingers.

For what concerns the other primary sensory tests, both the patient and all control participants correctly completed, with both hands, the single and the double tactile detection (both: 40/40), the pain, proprioception, and vibration assessments (all: 10/10). The only elementary sensory task slightly below 100% accuracy in the control sample was the temperature discrimination with the right hand (*M* = 9.92 ± 2.28, vs. patient: 10/10) (see Table 2).

**Table 2:** CP’s and healthy controls’ scores at the experimental tasks.

Test	CP’S results	Controls (mean)
Elementary sensory data		
Single touch detection		
Right hand	20/20	20/20
Left hand	20/20	20/20
Double tactile detection		
Right hand	20/20	20/20
Left hand	20/20	20/20
Pain		
Right hand	10/10	10/10
Left hand	10/10	10/10
Temperature		
Right hand	10/10	9.92/10
Left hand	10/10	10/10
Vibration		
Right hand	10/10	10/10
Left hand	10/10	10/10
Proprioception		
Right hand	10/10	10/10
Left hand	10/10	10/10
Intermediate somatosensory functions		
Weight		
Right hand	8/8	7.83 ± 0.38
Left hand	8/8	7.89 ± 0.32
Texture		
Right hand	8/8	8/8
Left hand	8/8	8/8
Size		
Right hand	8/8	8/8
Left hand	8/8	8/8
Materials		
Right hand	6/10^*^	9.33 ± 0.87
Left hand	8/10	9.33 ± 1.32
Bidimensional geometrical shapes		
Right hand	5/8^*^	7.89 ± 0.32
Left hand	8/8	7.61 ± 0.50
Tridimensional geometrical shapes		
Right hand	7/8^*^	8/8
Left hand	8/8	7.89 ± 0.32
Meaningless shapes		
Right hand	7/12*	11.50 ± 0.51
Left hand	11/12	11.33 ± 0.84
Real object recognition		
Real object comparison (right hand)		
Accuracy	50/64^*^	63 ± 1
Exploration time first stimulus (in seconds)	43.65 ± 14.48^*^	3.32 ± 1.1
Exploration time second stimulus (in seconds)	26.49 ± 16.84^*^	2.61 ± 0.6

Mean ± standard deviation values are reported for control participants. ^*^Significantly different from controls at Crawford–Howell *t*-test (*p* < 0.01).

As the processing of elementary sensory information was intact, we proceeded with the investigation of the intermediate level of elaboration.

### 3.2 Hylognosis and morphognosis

CP correctly responded to every weight comparison (8/8 for each hand; controls score: *M* right = 7.83 ± 0.38; *M* left = 7.89 ± 0.32), every texture comparison (8/8 for each hand, as controls), and every size comparison (8/8 for each hand, as controls).

Regarding materials, she named six materials out of 10 with the right hand (plastic, foam rubber, rubber, paper, cotton, and fabric), and eight materials out of 10 (plastic, wood, foam rubber, paper, rubber, cotton, polystyrene, and fabric) with the left hand. These scores were significantly different from controls (*M* right hand = 9.33 ± 0.87; *M* left hand = 9.33 ± 1.32) for the right hand [*t*(8) = −3.63, *p* = 0.003], but not for the left hand [*t*(8) = −0.96, *p* = 0.183].

Concerning shapes recognition, CP was selectively impaired only with the right hand in exploring bidimensional [score: 5/8; controls score: *M* right = 7.89 ± 0.32; *M* left = 7.61 ± 0.50; *t*(17) = −8.79, *p* < 0.001] and tridimensional geometrical shapes [score: 7/8; controls score: *M* right = 8; *M* left = 7.89 ± 0.32; *t*(17) = −Inf, *p* < 0.001]. Moreover, she was significantly less accurate than controls (*M* right = 11.50 ± 0.51; *M* left = 11.33 ± 0.84) on the meaningless shapes test with the right hand [score: 7/12, *t*(17) = −8.58, *p* < 0.001] but not with the left hand [score: 11/12, *t*(17) = −0.38, *p* = 0.35].

### 3.3 Real object recognition

#### 3.3.1 Nottingham sensory test—stereognosis subtest

In the *Nottingham Sensory Test*, the patient scored 13/20 with the right hand and 19/20 with the left hand; with the left hand she only mistook the flannel for a cloth, but she properly described the material.

#### 3.3.2 Naming of objects

In the naming test, with the right hand, CP correctly named 3 objects out of 12 (fork, teaspoon, and watch), while referring not to being sure about the answers; with the left hand, she correctly named all 12 objects. She also correctly named all the objects through the visual modality (12/12). A paired-sample *t*-test showed that exploration time was significantly longer [*t*(11) = 9.81, *p* < 0.01] for the right hand (*M* = 44.7 ± 13.52 s) than for the left hand (*M* = 5.30 ± 2.71 s).

#### 3.3.3 Drawing an object after haptic exploration

**Figure 2:**
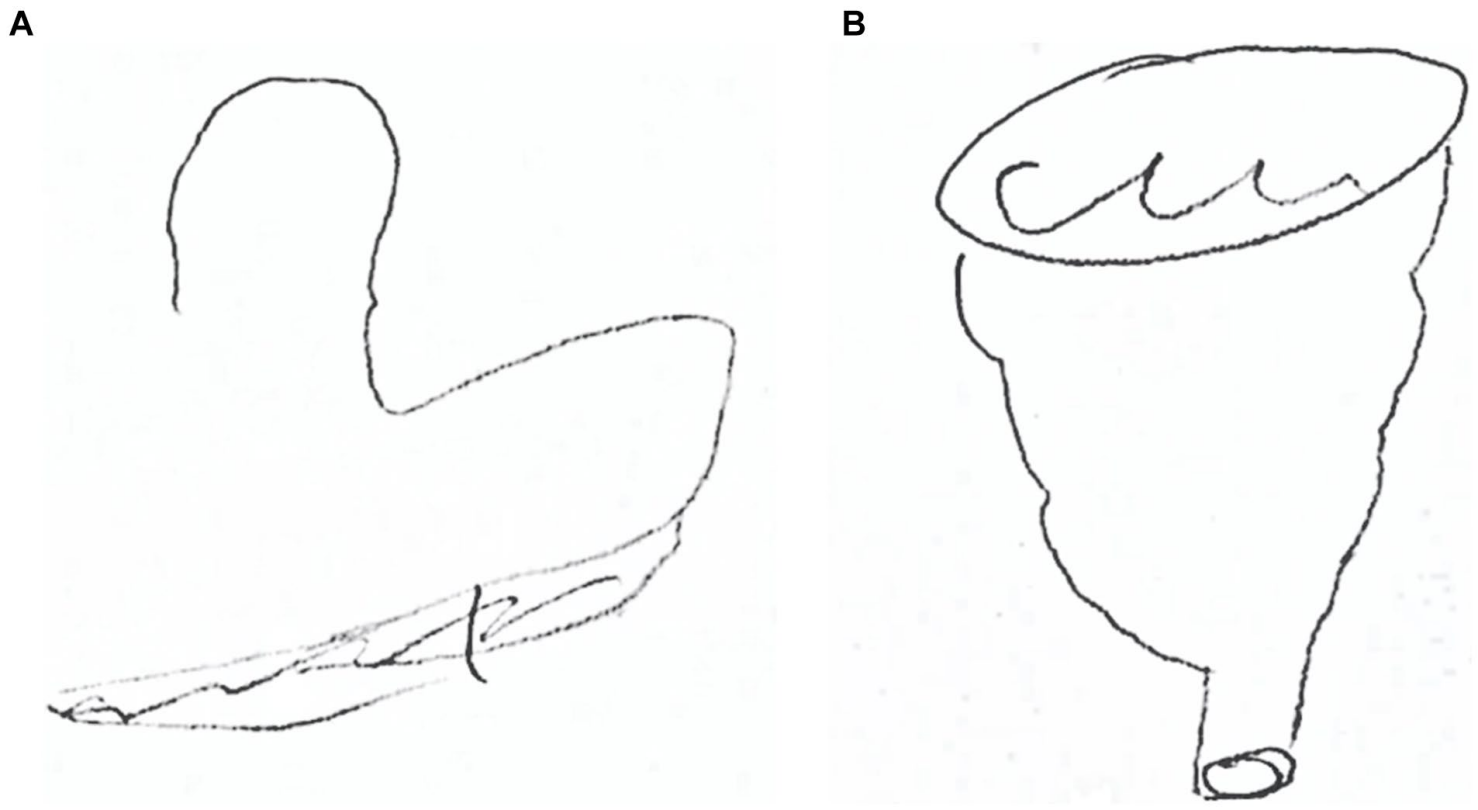
CP’s drawing of a funnel after right-hand exploration **(A)** and after left-hand exploration **(B)**.

Figure 2 displays performance of patient CP in drawing the funnel after tactile exploration. When the exploration was performed with the right hand, the patient failed to reproduce the shape (see Figure 2A) and to name it. Conversely, she promptly denominated the object manipulated with her left hand and drew it properly (see Figure 2B).

#### 3.3.4 Haptic recognition test of meaningful objects

The patient was both slower [first stimulus: 43.65 ± 14.48; *t*(17) = 35.68, *p* < 0.001; second stimulus: 26.49 ± 16.84, *t*(17) = 38.73, *p* < 0.001] and less accurate [score: 50/64, *t*(17) = −12.66, *p* < 0.001] than controls (first stimulus: *M* = 3.32 ± 1.1; second stimulus: 2.61 ± 0.6; accuracy: *M* = 63 ± 1.1) in exploring real objects. Performance of CP in each type of pair was significantly different from the control group (all comparisons *p* < 0.001). In the same category condition, she scored 7/16 [controls: *M* = 15.44 ± 1.10; *t*(17) = −7.49, *p* < 0.001], while she scored 14/16 for non-related [controls: *M* = 16.00; *t*(17) = -Inf, *p* < 0.001] and semantic-related objects [controls *M* = 15.94 ± 0.24; *t*(17) = −8.03, *p* < 0.001] and 15/16 for identical objects [controls: *M* = 15.94 ± 0.24; *t*(17) = −3.90, *p* = 0.001].

During this task, the patient tried to name the object, although it was not requested. In the few cases in which she succeeded in the identification (19%), it seemed that she based on the information derived by the qualities of the object (hylognosis) (see Table 3).

**Table 3:** CP’s comments while manipulating real objects.

Objects	Comment
Screw	*“It is cold, and I feel a knurling, so it could be a screw.”*
Two identical bottle cups	*“I do not know what these objects are, but they are both hollowed out in the center, so they could be the same.”*
Tea bag	*“It feels like a fabric from the texture, but it does not make that sound.”*
Two different types of erasers	*“This could be the same texture and material than the previous one, but it is smaller. I do not know what it is, but I think they could be the same object.”*

### 3.4 Follow-up

Considering the neuropsychological assessment, CP showed a generally very slow worsening of her global cognitive functioning as demonstrated by the lower score at the MMSE in 2023 (26.15 vs. 30 at the first assessment). Furthermore, apraxia deteriorated in the right hand (score 20/72 vs. 55/72 at first assessment) and also extended to the left hand (score 49/72 vs. 65/72 at first assessment; cut off = 62; Tessari et al., 2013). The Nottingham Sensory test for the left hand diminished only by one point, resulting in 18/20 (vs. 19/20 at first assessment), still within the normal range (Zamarro-Rodríguez et al., 2021).

#### 3.4.1 Right-hand experimental tactile evaluation

At 2-year follow-up, the right-hand apraxia and limb rigidity had worsened to the extent to prevent objects manipulation and the execution of most of the tests (*Nottingham Sensory Test*, bidimensional geometrical shapes, tridimensional geometrical shapes, and meaningless shapes). We evaluated the patient’s sensory perception abilities of the right hand at elementary and intermediate levels (see Table 4). Ability of CP to detect single (score: 40/40) and double stimuli (score: 40/40) was intact, as well as her ability to discriminate weight (score: 8/8) and temperature (score: 10/10). However, the patient showed lower scores in pain discrimination [score: 6/10; *t*(17) = −Inf, *p* < 0.001], vibration [score: 9/10; *t*(17) = −Inf, *p* < 0.001], proprioception [score: 5/10; *t*(17) = −Inf, *p* < 0.001], texture discrimination [score: 7/8; *t*(17) = −Inf, *p* < 0.001], and size [7/8; *t*(17) = −Inf, *p* < 0.001], compared to controls. These findings confirmed an extension of the disorder also to the basic and intermediate sensory tactile abilities, except tactile stimuli detection, temperature, and weight discrimination.

**Table 4:** CP’s scores at the experimental tasks at the first assessment (2021) and at the follow-up (2023).

Test	CP’S results in 2021	CP’S results in 2023
Elementary sensory data		
Single touch detection		
Right hand	20/20	20/20
Left hand	20/20	20/20
Double tactile detection		
Right hand	20/20	20/20
Left hand	20/20	20/20
Pain		
Right hand	10/10	6/10
Left hand	10/10	10/10
Temperature		
Right hand	10/10	10/10
Left hand	10/10	10/10
Vibration		
Right hand	10/10	9/10^*^
Left hand	10/10	10/10
Proprioception		
Right hand	10/10	5/10^*^
Left hand	10/10	10/10
Intermediate somatosensory functions		
Weight		
Right hand	8/8	8/8
Left hand	8/8	8/8
Texture		
Right hand	8/8	7/8^*^
Left hand	8/8	8/8
Size		
Right hand	8/8	7/8^*^
Left hand	8/8	8/8
Materials		
Right hand	6/10^*^	n.a.
Left hand	8/10	5/10^*^
Bidimensional geometrical shapes		
Right hand	5/8^*^	n.a.
Left hand	8/8	7/8
Tridimensional geometrical shapes		
Right hand	7/8^*^	n.a.
Left hand	8/8	7/8^*^
Meaningless shapes		
Right hand	7/12^*^	n.a.
Left hand	11/12	9/12^*^
Stereognosis subtest of NSA		
Right hand	13/20^*^	n.a.
Left hand	19/20	18/20

^*^Significantly different from controls at Crawford–Howell *t*-test (*p* < 0.01). n.a., not administrable.

#### 3.4.2 Left-hand experimental tactile evaluation

CP left hand appeared unimpaired at the first assessment.

At follow-up, all elementary sensory abilities were still intact (simple tactile detection: 40/40, pain 10/10, temperature 10/10, vibration 10/10, and proprioception 10/10) and hyloagnosia [weight 8/8, texture 8/8; materials 5/10, significantly different from controls, *t*(8) = −3.11, *p* = 0.07], together with size discrimination (score: 8/8). At the bidimensional geometrical shapes discrimination, CP scored 7/8, which was not significantly different from that of controls [M = 7.61 ± 0.50, *t*(17) = −1.19, *p* = 0.125]; conversely, the 7/8 score at the tridimensional geometrical shapes discrimination was different from the control sample [control M = 7.89 ± 0.32, *t*(17) = −2.71, *p* = 0.007]. She also lost 2 points as compared to the first assessment in the meaningless shapes test, scoring 9/12. Being this score significantly different from that of the controls [M = 11.33 ± 0.84, *t*(17) = −2.70, *p* = 0.007], it determined the onset of morphoagnosia in the left hand (see Table 4).

### 3.5 Anatomical data

Across the 3 years, no tumor relapse or modifications were reported in the area affected by the removal of the oligodendroglioma, i.e., the superior parietal gyrus, the cuneus, the precuneus, and the superior occipital gyrus in the right hemisphere.

**Figure 3:**
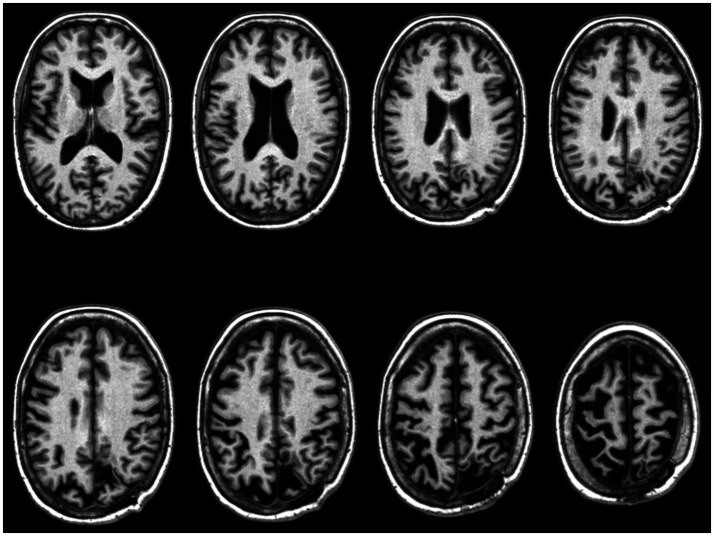
Axial view of CP’s T1-weighted brain scans acquired in 2023 (neurological convention).

On the other hand, in 2023, the neuroradiologist reported the presence of millimetric hypertensive foci in the subcortical bihemispheric white matter, markedly enlarged ventricles, and cortical sulci, especially at the vertex, asymmetrical due to greater dimensions at the left vertex (Figure 3). The linear hyperintense cortical signal alteration in the long TR sequences was unchanged, with a radiological profile compatible with the clinical suspicion of CBD.

Finally, 3 years from the clinical onset (2023), the DaTSCAN became positive, showing reduced integrity of the presynaptic dopaminergic system in the left putamen.

## 4 Discussion

Here, we presented the case of a 55-year-old woman who showed, at the first assessment, a severe unilateral right-hand impairment in tactile object recognition and apraxia, in the absence of elementary sensory or cognitive deficits. When assessing the intermediate level of tactile processing, CP showed intact abilities in distinguishing texture, weight, and size with both hands, but she was impaired in naming materials and discriminating geometrical and meaningless shapes with her right hand. These results are compatible with the presence of a unilateral right-hand morphoagnosia, with an almost entirely preserved ability to discriminate the qualities of the objects (i.e., hylognosis). To our knowledge, this is the third case described in the literature of a dissociation between morphognosis, significantly impaired, and an almost completely preserved ability to discriminate object qualities (i.e., hylognosis) (Saetti et al., 1999; Kubota et al., 2017). Moreover, while shape discrimination was impaired for both two- and three-dimensional geometric forms and meaningless shapes, size discrimination was intact, suggesting that shape and size perception can be dissociated, as hypothesized by the model proposed by Kubota et al. (2017).

Furthermore, the patient showed a deficit in real object recognition. Nevertheless, she could still recognize some objects. Indeed, CP showed an accuracy of 25% in the naming task and 19% in the meaningful objects haptic recognition test. This result suggests that the impairment at the apperceptive level does not completely prevent the identification of real objects, as suggested by Reed et al. (1996). We speculate that the intact hylognosis abilities allowed the identification process, as competence of CP in discriminating the qualities of objects was almost fully preserved.

Finally, it is important to point out that, given the presence of right-hand apraxia, the observed deficits in object recognition may have been due to a difficulty in tactile exploration (Valenza et al., 2001) or to a deficit in the integration of motor command (Dijkerman and de Haan, 2007). However, to overcome the influence of apraxia on the task of object recognition, the patient’s exploration was guided by the experimenter in both bi- and tridimensional geometric figure recognition. Despite this intervention, the patient was still unable to identify the objects. Based on the above-reported neuropsychological and neurological data and considering the clinical evolution of the disease, in particular the right limb rigidity, the worsening of apraxia, the appearance of morphoagnosia in both hands, and the positivity of the last DATsCAN, we propose that the patient presented with CBS characterized by a deficit in object recognition at the onset.

The neural correlates in this patient are very complex as she presented both a left frontoparietal atrophy and a lesion outcome of a right parietal oligodendroglioma removal. The left atrophy observed at the first MRI, involving the precentral and postcentral gyri, is compatible with the neural correlates of contralateral tactile object recognition reported in the literature (Crutch et al., 2005; Hömke et al., 2009; Kubota et al., 2017). Additionally, as the worsening of the right-hand symptoms and the appearance of morphoagnosia and ideomotor apraxia in the left hand were not associated with a relapse of the tumor but with a progression of the left hemisphere atrophy, we assume it is improbable that the oligodendroglioma was the cause of the deficit of tactile recognition. The longitudinal follow-up of our patient allows us to discuss the implications of our results for the evaluation of CBS as well.

The diagnosis of CBS is coherent with the reported progressive left atrophy in the precentral and postcentral gyri (Whitwell et al., 2010). Furthermore, our case aligns with the cognitive profile described by Jütten et al. (2014) in patients with right-side CBS onset. Indeed, as in their cases, CP remained stable throughout the cognitive domains while getting worse in apraxia and morphoagnosia, and she showed brain atrophy in frontoparietal areas contralateral to the affected hand (i.e., the right hand) in the initial stage of the disease. Moreover, right-hand apraxia was characterized by progressive clumsiness of movements and difficulties in finger dexterity as already observed in other CBS cases (Stamenova et al., 2009).

There are a few studies that systematically investigated the symptoms of CBS, and only recently there is more interest in a better definition of the associated sensorimotor deficits (Rinne et al., 1994; Belfor et al., 2006; Hu et al., 2009; Matsuda et al., 2020). Indeed, as previously stated in the introduction, although “cortical sensory loss” is one of the diagnostic criteria for CBS (Armstrong et al., 2013), only one study provided a clear clinical definition (Matsuda et al., 2020). Interestingly, in Matsuda’s study, although the studied patients showed sensorimotor impairments, none of them presented shape or texture discrimination deficits, nor TOR impairments. Our patient showed the opposite pattern, that is, morphoagnosia, without hyloagnosia, and a deficit in real object recognition. To the best of our knowledge, this is the first described case of CBS with morphoagnosia at the onset.

## 5 Conclusion

To conclude, we here have illustrated a case of neurodegenerative disease with a deficit in object recognition at the illness onset. Our patient also presented with a complex neuroanatomical pattern due to the coexistence of a tumor in the right hemisphere and a progressive atrophy in the left hemisphere. Both the behavioral and anatomical evidence made the diagnosis complex, requiring a detailed exploration of the symptoms and a repeated clinical follow-up. We deeply investigated the somatosensory competencies of CP, applying hierarchical procedures from the elementary to the higher cognitive functions. Compared with other cases of CBS reported in the literature, our patient showed a different profile, in the absence of elementary sensory deficits, presenting with a morphognosic disorder. Her neuropsychological profile contributes to a better understanding of the complex model of object recognition and indicates the relevance of performing an early and extensive assessment of object recognition abilities in patients with neurodegenerative diseases such as CBS.

As stated by previous authors (Jütten et al., 2014), given the CBS asymmetric onset, with subsequent different patterns of pathology progression, it could be crucial to study CBS cases beginning with the examination of the side of the body first affected. This could help in the comprehension of the cognitive evolution of patients with CBS, improving diagnosis and prognosis. At the same time, given the frequent presence of sensorimotor deficits in CBS, their assessment is essential, and it needs to be standardized across studies. Our study demonstrated that, if thoroughly investigated, morphoagnosia can emerge as one of the early symptoms to monitor for CBS diagnosis.

## Data availability statement

The raw data supporting the conclusions of this article will be made available by the authors, without undue reservation.

## Ethics statement

The studies involving humans were approved by Comitato Etico Milano Area 3 and Comitato Etico Università di Pavia. The studies were conducted in accordance with the local legislation and institutional requirements. The participants provided their written informed consent to participate in this study. Written informed consent was obtained from the individual(s) for the publication of any potentially identifiable images or data included in this article.

## Author contributions

LF: Data curation, Formal analysis, Investigation, Visualization, Writing – original draft, Writing – review & editing. SB: Conceptualization, Validation, Writing – review & editing. MS: Formal analysis, Writing – review & editing. GG: Writing – review & editing. MG: Conceptualization, Methodology, Project administration, Supervision, Writing – review & editing. GB: Conceptualization, Resources, Supervision, Writing – review & editing.

## Funding

The authors declare that no financial support was received for the research, authorship, and/or publication of this article.
